# Sclerosing bone dysplasias: a pictorial essay

**DOI:** 10.1590/0100-3984.2024.0058-en

**Published:** 2025-01-06

**Authors:** Vinicius de Almeida Cavalcante Galdino, Marcelo Mantiolhe Martins, Vinícius Neves Marcos, Gabriel Fernandes Gonçalves, Rafaela Gonçalves Dias, Daniela Rambaldi Mileti

**Affiliations:** 1 Department of Radiology, Hospital Universitário da Universidade Federal de Juiz de Fora (HU-UFJF), Juiz de Fora, MG, Brazil; 2 Universidade Federal de Juiz de Fora (UFJF), Juiz de Fora, MG, Brazil

**Keywords:** Bone diseases, developmental, Hyperostosis, Osteosclerosis, Displasias ósseas, Hiperostose, Esclerose

## Abstract

Sclerosing bone dysplasias encompass abnormalities in bone density, divided into hereditary and nonhereditary forms. Primarily diagnosed through radiography, they are often incidental findings. Among the hereditary forms, the following stand out: osteopetrosis, osteopoikilosis, multiple diaphyseal sclerosis (ribbing disease), osteopathia striata, and Camurati-Engelmann disease. Among the nonhereditary forms, intramedullary osteosclerosis and melorheostosis present specific radiographic characteristics. The main differential diagnoses include osteoblastic metastases, tuberous sclerosis, and renal osteodystrophy, requiring careful differentiation because of their similarities.

## Abstract (pt)

As displasias ósseas esclerosantes abrangem anormalidades na densidade óssea, divididas em hereditárias e não hereditárias. Diagnosticadas principalmente por radiografia, muitas vezes são achados incidentais. Entre as formas hereditárias destacam-se a osteopetrose, a osteopoiquilose, a esclerose diafisária múltipla, a osteopatia estriada e a doença de Camurati-Engelmann. Entre as formas não hereditárias, a osteosclerose intramedular e a melorreostose apresentam características radiográficas específicas. Diferenciais importantes incluem as metástases osteoblásticas, a esclerose tuberosa e a osteodistrofia renal, exigindo diferenciação cuidadosa em razão das suas semelhanças.

## INTRODUCTION

Sclerosing bone dysplasias (SBDs) are abnormalities resulting from focal or diffuse increases in bone density, and, because they present characteristic features, they can be diagnosed by conventional radiography^(1)^. Radiologists should be familiar with the typical radiological findings to differentiate SBD from other causes of bone sclerosis. This pictorial essay aims to illustrate and differentiate among SBDs, as well as to exemplify some differential diagnoses that are important for clinical practice.

## HEREDITARY SBD

Hereditary SBD can be symptomatic, in which case it will be diagnosed in childhood, or asymptomatic and diagnosed late, in adulthood^(1)^.

### Osteopetrosis

Osteopetrosis is a dysplasia of the spongy layer characterized by decreased osteoclast activity, which results in changes in bone remodeling, increasing bone thickness and altering the bone morphology, thus increasing the risk of fractures^(2)^. The autosomal recessive subtype is characterized by premature death, whereas patients with the autosomal dominant subtype can be either asymptomatic or present complications such as fractures, osteomyelitis, and cranial nerve injuries^(2)^.

**Figure 1:**
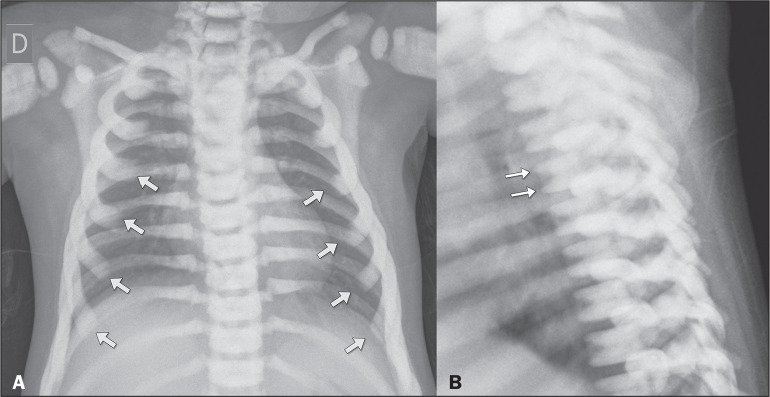
Chest X-rays of a 16-monthold patient with the autosomal recessive form of osteopetrosis who underwent the examination because of suspected pneumonia. Note the diffuse increase in bone density, with a loss of cortical-medullary differentiation. In the posteroanterior view (A), note the widening of the costochondral junctions (arrows). The lateral view (B) shows the characteristic “sandwich vertebrae” appearance, resulting from the accumulation of bone in the superior and A B inferior vertebral endplates (arrows).

Radiological changes in osteopetrosis are marked by diffuse increased bone density with a loss of corticomedullary differentiation, as well as by recent or healing fractures. Other characteristic changes include widening of the costochondral junctions (Figure 1); characteristic metaphyseal widening (Erlenmeyer flask deformity); bone-within-bone and alternating radiolucent/radiodense metaphyseal lines; and diffuse vertebral endplate sclerosis, also known as “sandwich vertebrae” (Figure 1).

### Osteopoikilosis

**Figure 2:**
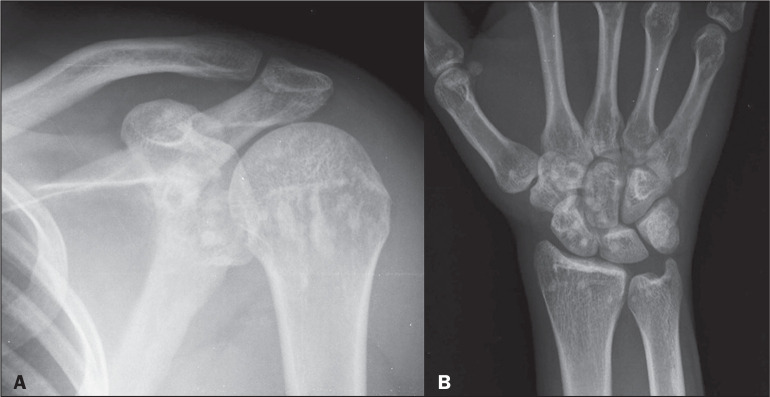
Shoulder and wrist X-rays of a 21-year-old patient with a history of motor vehicle accident trauma. Note the multiple foci of increased bone density in the humeral head and glenoid on the shoulder X-ray (A), as well as in the bony elements in the right wrist (B), a fi nding A B typical of osteopoikilosis.

Osteopoikilosis, or disseminated condensing osteopathy, is an endochondral ossification disorder involving the secondary spongiosa, resulting in focal deposits of compact bone with the appearance of enostoses (bone islands). It presents as multiple sclerotic foci, in some cases with spicules that blend with the surrounding trabeculae, in the shape of a flame or a blade of grass^(3)^. On computed tomography (CT), the characteristic appearance is that of multiple bone islands of different sizes, deposited at the ends of short tubular bones, tarsal bones, carpal bones, and pelvic bones, as well as in the metaepiphyseal regions of long bones^(1,3,4)^, as shown in Figure 2. In patients who are at increased risk for sclerotic bone metastases, evaluation by bone scintigraphy may be necessary, given that the bone islands do not demonstrate increased uptake.

### Osteopathia striata

**Figure 3:**
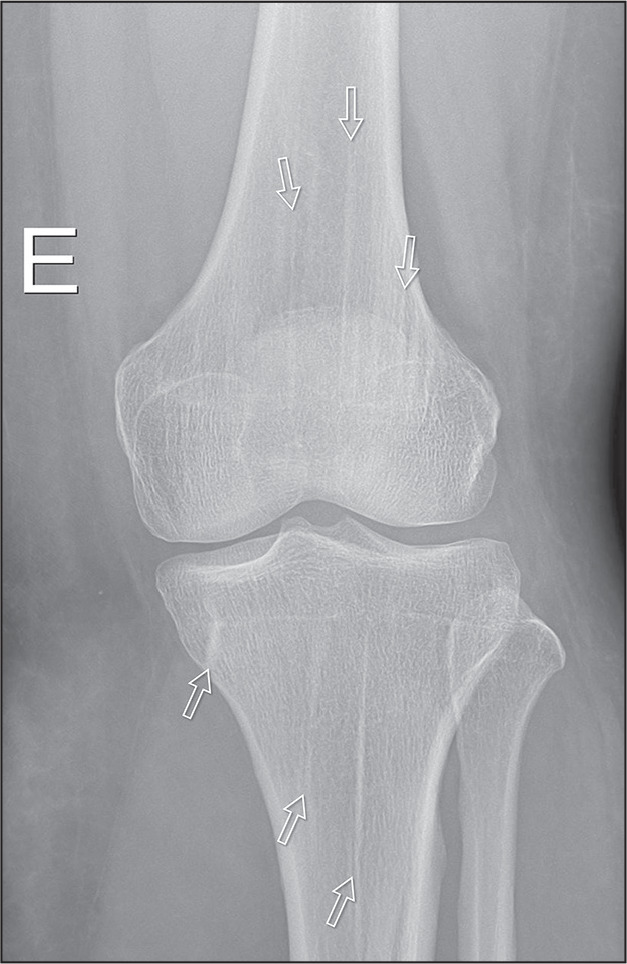
X-ray of the left knee of a 25-year-old patient with a history of trauma due to being run over by a motor vehicle. Note the vertical radiopaque striations in the bone marrow of the distal femur and proximal tibia, with no other changes in the bone marrow or cortex, findings characteristic of osteopathia striata.

Osteopathia striata is a secondary spongy bone disorder caused by an imbalance between bone formation by osteoblasts and resorption by osteoclasts, leading to increased formation or limited resorption. It does not cause physical abnormalities and is diagnosed incidentally on imaging examinations^(3)^. It is characterized by dense linear striations in the diaphyses and metaphyses of long tubular bones. The striations run parallel to the long axis of the bone and are typically seen in areas of rapid growth, such as the femur (Figure 3). In the iliac bones, the striations may have a fanshaped appearance due to their growth patterns^(3)^.

### Ribbing disease

**Figure 4:**
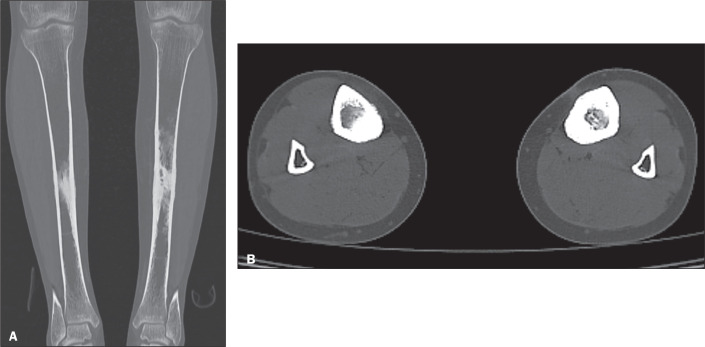
Coronal and axial CT scans of the lower limb joints (A and B, respectively) of a 35-year-old patient with a history of chronic pain in the anterior aspect of the tibia. Note the thickening of the cortical bone, involving both the periosteal and endosteal surfaces, in addition to sclerosis of the medullary canal in the middle third of the bilateral tibia. The changes described determine narrowing of the medullary canal. In diaphyseal sclerosis, bilaterally asymmetrical or unilateral involvement of the tibia is typical, with periosteal and endosteal thickening, sparing the metaphyses and epiphyses.

**Figure 5:**
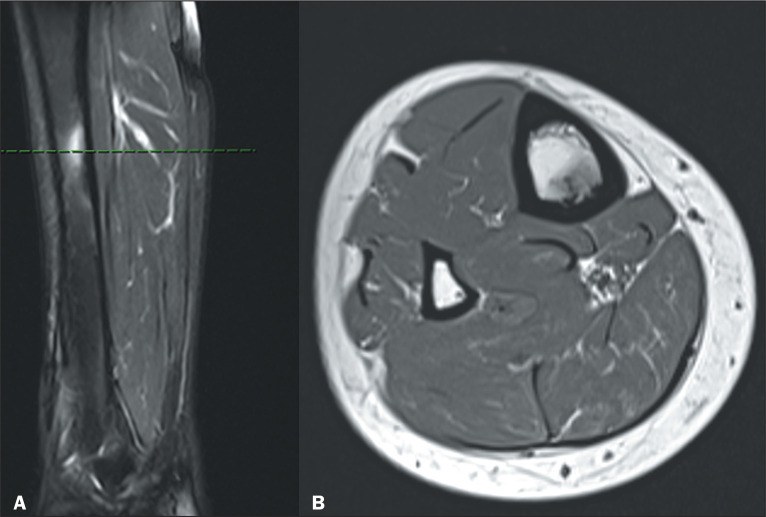
Magnetic resonance imaging of the lower limb of the patient in Figure 4, sagittal T2-weighted image with fat saturation (A) and axial T1-weighted image without fat saturation (B, at the level of the green dotted line in image A). Note the thickening of the cortex in the middle third of the tibia (better characterized in B), presenting signal alteration with a pattern of edema in the adjacent bone marrow (demonstrated in A), without soft tissue involvement.

Hereditary multiple diaphyseal sclerosis, or ribbing disease, is a disorder of intramembranous ossification. It manifests after puberty and may progress slowly or stabilize. Examinations demonstrate cortical thickening, involving the periosteum and endosteum of the diaphyseal portion of long bones, especially the femur and tibia, sparing the epiphyses (Figures 4 and 5). It may progress to narrowing of the medullary canals^(3,5)^. It is a diagnosis of exclusion, the main differential diagnoses being osteosarcoma, osteoid osteoma, osteomyelitis, stress fracture, and Camurati-Engelmann disease^(5)^.

### Camurati-Engelmann disease

Camurati-Engelmann disease is characterized by changes in the skull and in the diaphyses of long tubular bones. It manifests as bone pain, reduced muscle mass, and hypotonia of the lower limbs. Cranial hyperostosis and fusiform bone enlargement/sclerosis of long bones are observed, together with irregular cortical thickening of the diaphyses, as well as hyperostosis extending to the periosteum and endosteum. The hyperostosis is typically bilateral but may be asymmetrical^(6)^.

## NONHEREDITARY SBDs

### Intramedullary osteosclerosis

**Figure 6:**
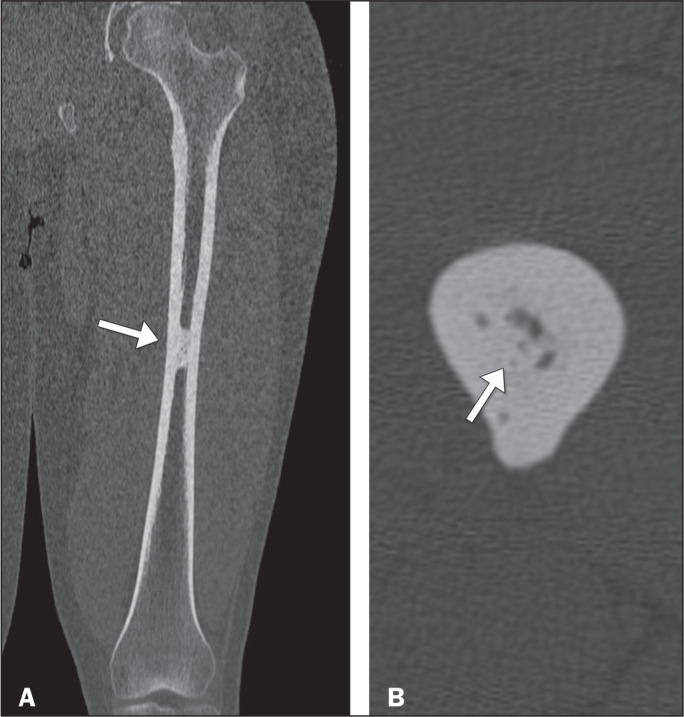
Coronal (A) and axial (B) CT scans of the lower limbs of a 32-year-old patient after pedestrian versus motor vehicle accident-related trauma. In A and B, note the sclerosis limited to the medullary cavity of the tibial diaphysis (arrows), shown in the coronal and axial planes, respectively, and the lack of thickening of the cortical bone, findings typical of intramedullary osteosclerosis.

Intramedullary osteosclerosis is an endosteal bone formation with diaphyseal sclerosis in the long bones of adults, and the involvement is asymmetric. Its main symptom is chronic mechanical pain in the diaphyses of long bones^(7)^. It is characterized by increased bone formation in the medullary space of long bones (the tibia, fibula, and femur), without cortical thickening or periosteal reaction (Figure 6). There can be edema of the soft tissues adjacent to the lesion. Bone scintigraphy shows intense uptake in the affected regions, allowing the usual distribution to be characterized, helping differentiate intramedullary osteosclerosis from other sclerosing dysplasias^(3)^. The diagnosis tends to be incidental, and the differential diagnoses include stress fractures, osteomyelitis, metabolic disorders, endocrine disorders, and bone-forming tumors^(7)^.

### Melorheostosis

**Figure 7:**
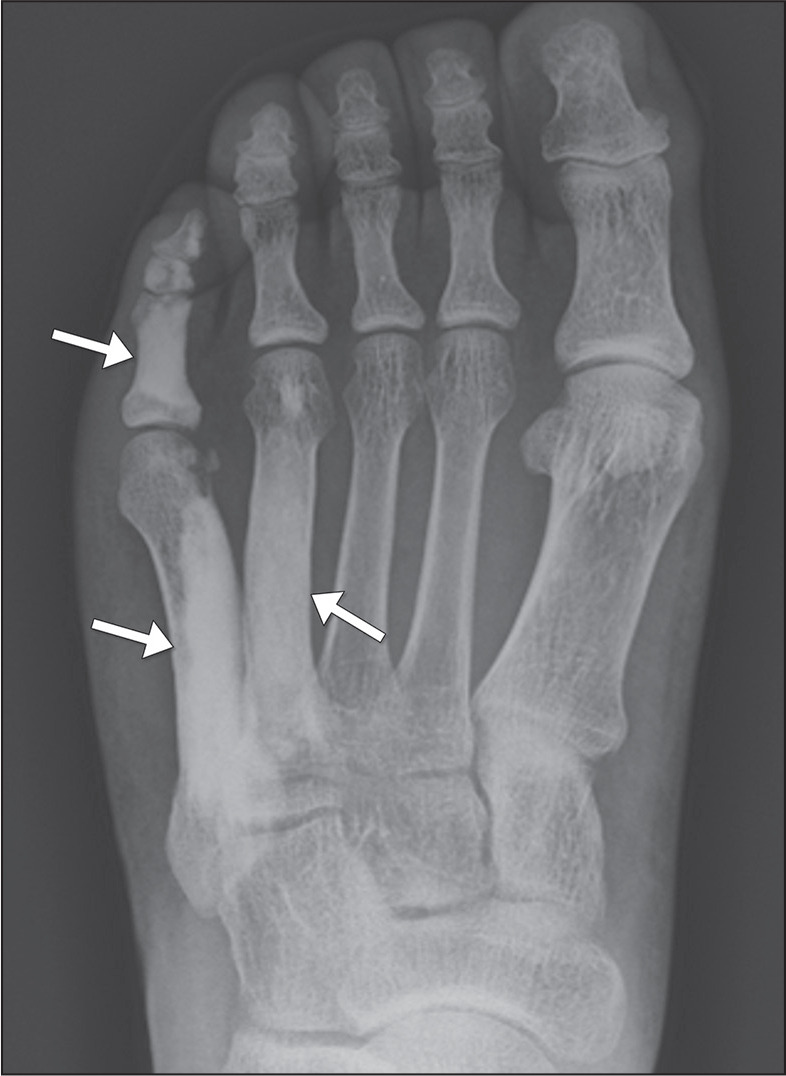
Anteroposterior X-ray of the left foot of a 39-year-old patient complaining of localized pain. The sclerotic changes involve the cortical and medullary bone (arrows) of the fourth and fifth metatarsals, as well as those of the phalanges of the fifth ray, wavy contours creating the “dripping candle wax” appearance typical of melorheostosis.

**Figure 8:**
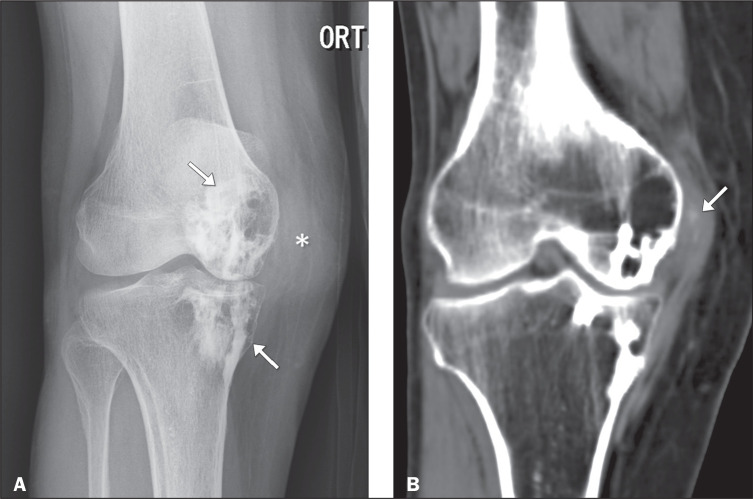
Anteroposterior X-ray of the knee (A) of a 75-year-old patient with a history of localized pain. Note the irregular sclerotic lesions (arrows) in the femoral condyle and medial tibial plateau, with a “dripping candle wax” appearance, typical of melorheostosis. CT scan (B) showing thickening and densification of the soft tissues adjacent to the medial femorotibial compartment (asterisk) involving the area of the medial collateral ligament, encompassing a focus of calcification that was better characterized on CT, another finding commonly seen in melorheostosis.

Melorheostosis, also known as Leri’s disease, is a mixed sclerosing bone dysplasia that disturbs endochondral and ossification, with a distribution that respects the dermatomes. Classically, the lesions are sclerotic, with cortical and medullary hyperostosis, resulting in wavy bone edges, a finding known as the “d ripping candle wax” sign (Figure 7), and the pattern tends to be segmental and unilateral, commonly associated with involvement of adjacent soft tissues, such as skin lesions and muscle atrophy^(1,3)^, as depicted on CT in Figure 8.

### Overlap syndromes

Overlap syndromes are nonhereditary dysplasias that present characteristics of two or more SBDs simultaneously. Various combinations have been described, the most common being that of melorheostosis, osteopoikilosis, and osteopathia striata. Because of the overlapping symptoms, overlapping syndromes can easily be confused with sclerotic metastases^(3)^.

## DIFFERENTIAL DIAGNOSES

The symptoms and radiographic features of SBDs can overlap with those of other conditions, whether metabolic or neoplastic, which must be excluded to proceed with appropriate management^(3)^. Some of the differential diagnoses are described below. For appropriate recognition by the radiology community, they can be distinguished from SBDs either by their prevalence, as in the case of osteoblastic metastases and renal osteodystrophy, or by their rarity, as in the case of tuberous sclerosis^(3)^.

### Renal osteodystrophy

**Figure 9:**
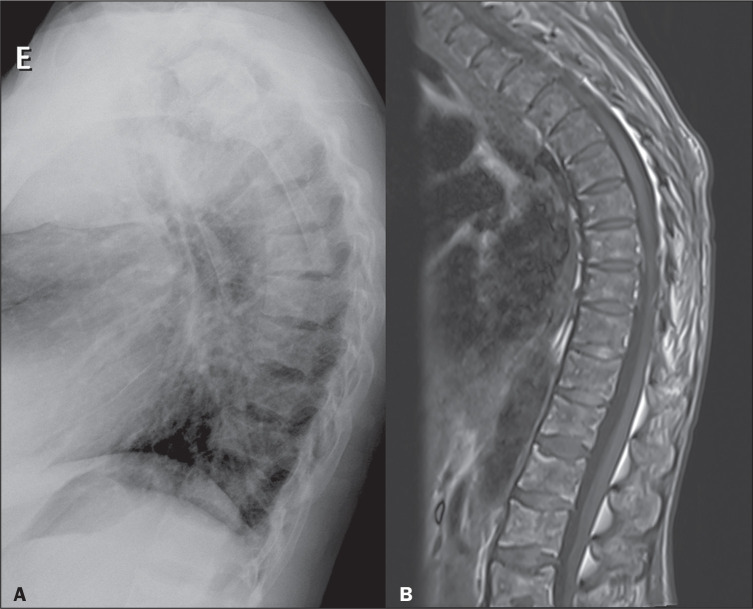
A 35-year-old patient with chronic kidney disease, requiring dialysis, and secondary hyperparathyroidism. A: Lateral chest X-ray showing diffuse increased bone density of the vertebral bodies, a fi nding consistent with renal osteodystrophy. B: Sagittal T1-weighted magnetic resonance imaging scan, without fat saturation, showing diffuse reduction of the bone marrow signal, without evident focal lesions, a finding that confirms the osteosclerosis in this patient with renal osteodystrophy.

Renal osteodystrophy refers to findings observed in the context of chronic kidney disease, presenting as osteomalacia and secondary hyperparathyroidism. Because of the anabolic effect of parathyroid hormone, the affected bone may present a diffuse increase in radiodensity, a condition known as diffuse osteosclerosis. In most cases, that is seen in the axial skeleton, where there is more trabecular bone than cortical bone (Figure 9), and it can lead to the development of an SBD. However, despite the increase in radiodensity, the bone is structurally weak and more prone to fractures^(6)^.

### Osteoblastic metastases

**Figure 10:**
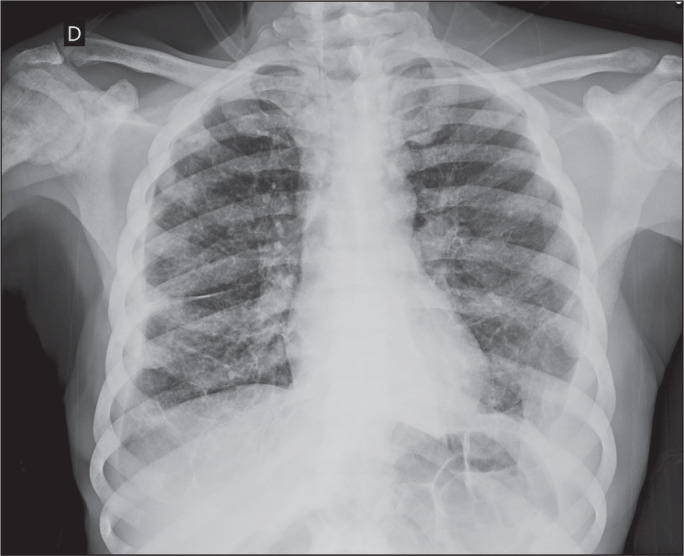
Chest X-ray obtained for investigation of anemia and wasting syndrome in a 63-year-old patient, showing a diffuse increase in bone density, sometimes with a heterogeneous appearance (best seen in the right humeral head), suggesting infiltrative sclerotic bone lesions throughout the bone structure. Subsequently, a diagnosis of prostate adenocarcinoma was made, confirming the suspicion of osteoblastic bone metastasis.

Osteoblastic metastases must be recognized in order to avoid diagnostic delays and to continue the necessary investigation to identify the primary tumor. The primary tumors most often associated with osteoblastic metastases include prostate carcinoma, breast carcinoma, pancreatic adenocarcinoma, carcinoid tumor, lymphoma, medulloblastoma, and neuroblastoma^(3)^. The clinical history, together with radiographic findings of infiltrative lesions (Figure 10), which can be accompanied by cortical erosion and soft tissue involvement, should alert to this diagnosis^(3)^.

### Tuberous sclerosis

**Figure 11:**
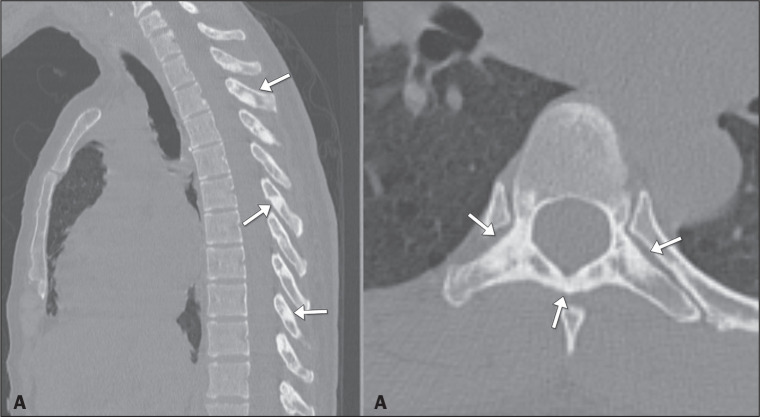
Chest CT in the sagittal and axial planes (A and B, respectively) of a 43-year-old patient with tuberous sclerosis, showing irregular sclerotic areas in the pedicles and posterior laminae of the thoracic and cervical vertebrae (arrows). Note the sclerotic changes in the pedicles, transverse processes, and posterior laminae (arrows in B).

Tuberous sclerosis is characterized by benign congenital tumors in multiple organs. Sclerotic bone lesions are the third most common imaging finding in patients with tuberous sclerosis and are therefore included in its diagnostic criteria. Radiologists should be aware of these bone changes to avoid diagnostic confusion, especially with osteoblastic metastases. On CT, sclerotic bone lesions resemble islands of bone within the medullary cavities of the bones, usually located in the vertebral bodies and posterior elements of the spine, and can also be seen in the sacrum (Figure 11). Scintigraphy demonstrates an absence of tracer uptake, which distinguishes tuberous sclerosis from osteoblastic metastasis^(8)^.

### Paget’s disease of bone

**Figure 12:**
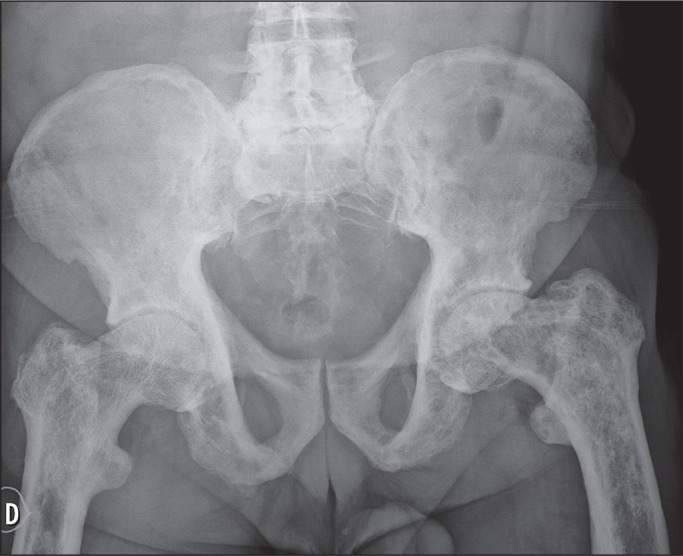
Anteroposterior X-ray of the pelvis of a patient with a confirmed diagnosis of Paget’s disease of bone, showing diffuse changes characteristic of this disease, such as changes in the morphology of the pelvis and femurs, in addition to thickening of the cortical bone, with coarse, irregular medullary trabeculae.

Paget’s disease of bone is a chronic osteometabolic condition that results in excessive bone remodeling. There is an initial phase of bone resorption, with a predominance of osteolytic lesions, followed by disordered bone formation, characterized by coarse trabeculae and bone sclerosis (Figure 12), making the bones fragile and susceptible to fractures^(9)^. The changes depend on the location and evolutionary phase of the disease, with the most commonly affected bones being the pelvis, spine, skull, and proximal long bones^(10)^.

To facilitate the recognition of and distinctions between the main radiographic findings of SBDs, Table 1 highlights characteristics suggestive of each dysplasia, and Table 2 presents the most common differential diagnoses of SBDs.

**Table 1:** Main radiographic findings of SBDs.

Dysplasia		Classification	Typical radiographic features
Osteopetrosis Osteopoikilosis Osteopathia striata Ribbing disease Camurati-Engelmann disease Intramedullary osteosclerosis Melorheostosis		Hereditary Hereditary Hereditary Hereditary Hereditary Nonhereditary Nonhereditary	Diffuse increase in bone density with loss of corticomedullary differentiation, fractures, widening of costochondral junctions, Erlenmeyer flask deformity, bone-within-bone appearance, alternating clear/dense metaphyseal lines, and “sandwich vertebrae” sign Multiple bone islands: small round or oval sclerotic foci in the bone marrow, typically with irregular contours, and no cortical destruction or periosteal reaction Dense linear striations in the diaphyses and metaphyses of tubular long bones, lying parallel to the long axis of the bone in areas of rapid growth, with a fan-shaped appearance in the iliac bones in some cases Cortical thickening, involving the periosteum and endosteum of the diaphyseal portion of long bones, sparing the epiphyses, together with narrowing of the medullary canals in some cases Cranial hyperostosis, fusiform bone enlargement, and sclerosis of long bones, together with irregular cortical thickening of the diaphyses extending to the periosteum and endosteum, typically bilateral but asymmetric in some cases Increased bone formation in the medullary space of long bones, without cortical thickening or periosteal reaction, with edema of the adjacent soft tissues in some cases Sclerotic lesions with cortical and medullary hyperostosis and wavy bone edges (“dripping candle wax” sign), typically segmental and unilateral, with involvement of the adjacent soft tissues in many cases

**Table 2:** Main differential diagnoses of SBDs.

Differential diagnosis			Typical radiographic features
Renal osteodystrophy Osteoblastic metastases Tuberous sclerosis Paget’s disease of bone	Pattern of diffuse osteosclerosis with a diffuse increase in bone marrow radiodensity, together with frequent fractures (structurally weak bone) Infiltrative sclerotic lesions that can be accompanied by cortical erosion and soft tissue involvement Medullary sclerotic foci (like bone islands), in many cases located in the vertebral bodies and posterior elements of the spine and sacrum An initial phase of bone resorption, with a predominance of osteolytic lesions, followed by a phase with disordered bone formation, characterized by coarse trabeculae and bone sclerosis, with frequent changes in morphology		
